# Validity of reduced radiation dose for localized diffuse large B-cell lymphoma showing a good response to chemotherapy

**DOI:** 10.1093/jrr/rrt122

**Published:** 2013-11-01

**Authors:** Keiichiro Koiwai, Shigeru Sasaki, Eriko Yoshizawa, Hironobu Ina, Ayumu Fukazawa, Katsuya Sakai, Takesumi Ozawa, Hirohide Matsushita, Masumi Kadoya

**Affiliations:** 1 Department of Radiology, Shinshu University, School of Medicine, 3-1-1, Asahi, Matsumoto, 390-8621, Japan

**Keywords:** diffuse large B-cell lymphoma, radiation dose, radiotherapy, chemotherapy

## Abstract

To evaluate the validity of a decrease in the radiation dose for patients who were good responders to chemotherapy for localized diffuse large B-cell lymphoma (DLBCL), 91 patients with localized DLBCL who underwent radiotherapy after multi-agent chemotherapy from 1988–2008 were reviewed. Exclusion criteria were as follows: central nervous system or nasal cavity primary site, or Stage II with bulky tumor (≥10 cm). Of these patients, 62 were identified as good responders to chemotherapy. They were divided into two groups receiving either a higher or a lower radiation dose (32–50.4 Gy or 15–30.6 Gy, respectively). There were no statistically significant differences between the lower and higher dose groups in progression-free survival, locoregional progression-free survival or overall survival. Adaptation of decreased radiation dose may be valid for localized DLBCL patients who show a good response to chemotherapy.

## INTRODUCTION

Combinations of radiotherapy and chemotherapy for localized aggressive non-Hodgkin lymphoma (NHL) have been widely accepted as standard treatment regimens. Involved-field radiotherapy (IFRT) after a short course of chemotherapy, such as cyclophosphamide, doxorubicin, vincristine and prednisone (CHOP), can yield good results in such cases [1].

There have been major advances in chemotherapy, and further progress has been made in recent years with the advent of molecular-targeted agents [2]. There have also been changes in radiotherapy. A radiation dose above 40 Gy was adopted in a landmark trial that elucidated the effectiveness of short-course CHOP followed by IFRT [1, 3]. Therefore, a radiation dose after a short course of chemotherapy has generally been administered at this level. However, experience with Hodgkin's lymphoma (HL) has indicated a relation between a radiation dose >35–40 Gy and the occurrence of considerable adverse events [3, 4]. Reduction of radiation dose has become one of the major concerns for both HL and NHL. The results of a recent randomized trial indicated that 30 Gy is sufficient for aggressive NHL [5]. However, the population in this trial was relatively heterogeneous, and response to chemotherapy was not considered in the assignment of the patients. Some retrospective studies have indicated that the radiation dose should be individualized according to initial tumor size or response to chemotherapy [6–8].

In this study, a retrospective review of patients with localized diffuse large B-cell lymphoma (DLBCL) who have undergone radiotherapy after chemotherapy in our facility was performed to evaluate the validity of a decrease in radiation dose for those showing a good response to chemotherapy.

## MATERIALS AND METHODS

All the patients with localized NHL who underwent radiotherapy after multi-agent chemotherapy between February 1998 and March 2008 in our facility were retrospectively reviewed. Patients who fulfilled the following criteria were selected from those patients: DLBCL, Ann Arbor Stage I or non-bulky (<10 cm) Stage II, and primary site other than central nervous system or nasal cavity. First, patients who were diagnosed as having diffuse large-cell lymphoma of B-cell origin (in the Working Formulation) or DLBCL (in the World Health Organization classification) were selected [9, 10]. Second, patients with bulky Stage II disease were excluded because they seemed better treated as having advanced disease [11]. Third, primary central nervous system and nasal cavity lymphomas were excluded because they have been considered as individual clinical entities [12–14]. Eventually, a total of 91 patients were selected for further analysis. We identified good responders to chemotherapy from among these 91 patients. A good responder to chemotherapy was defined as a patient who showed complete disappearance of all detectable disease at the end of the chemotherapy. Results of physical examinations and morphological imaging studies just after chemotherapy were examined to identify good responders to chemotherapy. Functional imaging studies were not taken into account in the identification. A total of 62 patients were identified, and selected as candidates for this research. To evaluate the validity of a decrease in radiation dose for the good responders to chemotherapy, they were divided into two groups—one with a higher radiation dose (>30.6 Gy) and another with a lower radiation dose (≤30.6 Gy). The characteristics of the patients in the two groups are shown in Table 1. All of the patients provided informed consent.

**Table 1.** Characteristics of the group with higher radiation dose (>30.6 Gy) and the group with lower radiation dose (≤30.6 Gy) showing a good response to chemotherapy

Characteristics	Higher dose group	Lower dose group
Number of patients	27	35
Age	22–82 (median: 63)	21–84 (median: 71)
Primary site		
Waldeyer's ring	11	20
Lymph node	8	8
Head and neck	3	3
Gastrointestinal	4	1
Others	1	3
Maximum tumor size (cm)	2.0–9.0 (median: 4.2)	2.0–8.0 (median: 4.0)
International prognostic index		
0/1	20	21
2/3	7	14
Chemotherapy regimen		
ACOP 3 cycles	4
5 cycles	1
MACOP-B 8 cycles		5
12 cycles	2
CHOP 3 cycles	10	16
6 cycles	2	4
R-CHOP 3 cycles	3	12
6 cycles	3
Radiation dose (Gy)	32–50.4 (median: 40)	15–30.6 (median: 30)
Fraction size (Gy)		
1.5	4	28
1.8	17	6
2.0	6	1

CHOP = cyclophosphamide, doxorubicin, vincristine, prednisone; R-CHOP = rituximab, cyclophosphamide, doxorubicin, vincristine, prednisone; ACOP = doxorubicin, cyclophosphamide, vincristine, prednisone; MACOP-B = methotrexate, doxorubicin, cyclophosphamide, vincristine, prednisone, bleomycin.

Although chemotherapy regimens varied, CHOP or CHOP-like regimens were adopted for the majority of the patients. Rituximab was added to CHOP (R-CHOP) in the treatment for patients with CD20-expressing NHL after its approval by the Japanese medical insurance system in the year 2003. The lower dose group contained more patients who had undergone R-CHOP than the higher group did. The majority of the patients in both groups received short-course chemotherapy: 22 patients (81%) and 28 patients (80%) received short-course chemotherapy in the higher and lower dose groups, respectively.

In radiation therapy, a photon beam of 4 or 10 MV was mainly used. All patients were treated according to a once-daily fractionation schedule. The fraction size was 1.5–2.0 Gy, and 1.8 Gy was mainly chosen for the higher dose group and 1.5 Gy for the lower dose group. By consensus at our facility, a relatively high total dose (near 40 Gy) had been adopted up until the year 2000. This consensus has changed and the dose has been lowered since the year 2000. Of the 27 patients who started their therapy before 2000, 19 (70%) belonged to the higher dose group, whereas only 8 (23%) of the 35 patients who started their therapy after 2000 belonged to the higher dose group. Taking the consensus into consideration, the final individual dose was determined at the discretion of the attending physician. Although the radiation dose was not necessarily fixed, all patients consistently underwent IFRT. The involved field was defined as the regional area including the primary lesion and the involved nodes (determined by pretreatment evaluation) and adjacent uninvolved nodes.

Pretreatment evaluation necessarily included a physical examination, bone marrow biopsy and computed tomography (CT) scan. Positron emission tomography (PET) was included in the later years of the study. Posttreatment evaluation was performed in the same manner as that prior to treatment. The International Working Group response criteria were adopted in posttreatment evaluation [15, 16].

Relapse in any region or death due to any cause was counted as an event in progression-free survival analysis. Relapse in the irradiated area or death due to any cause was counted as an event in locoregional progression-free survival analysis. Death due to any cause was counted as an event in overall survival analysis. The survival rates were calculated from the start of chemotherapy. The survival curves were calculated using the Kaplan–Meier method. Statistical analyses were performed by the log-rank test using JMP version 5.1.1 (SAS Institute Inc., Cary, NC, USA). In all analyses, *P* <0.05 was taken to indicate statistical significance.

## RESULTS

All patients eventually achieved complete remission (CR) in posttreatment evaluation. The median follow-up time was 74 months (range, 8–188 months). Relapse occurred in nine patients in the higher dose group. Two of the relapses initially occurred in the irradiated site. Nine patients also experienced relapse in the lower dose group. Only one of those relapses initially occurred in the irradiated site.

**Fig. 1.**
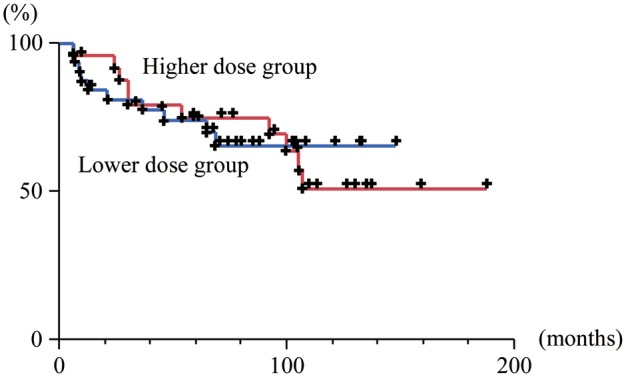
Progression-free survival curves of the group with higher radiation dose (>30.6 Gy) (red line) and the group with lower radiation dose (≤30.6 Gy) (blue line) among the good responders to chemotherapy.

The progression-free survival curves for the higher and lower dose groups are shown in Fig. 1: the five-year progression-free survival rates were 75% (95% confidence interval [CI]: 54–88) and 74% (95% CI: 56–97), respectively. The difference was not statistically significant (*P* = 0.99).

**Fig. 2.**
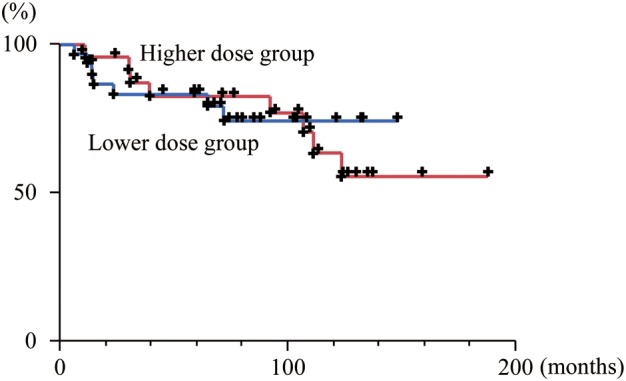
The locoregional progression-free survival curves of the group with higher radiation dose (>30.6 Gy) (red line) and the group with lower radiation dose (≤30.6 Gy) (blue line) among the good responders to chemotherapy.

The locoregional progression-free survival curves for the higher and lower dose groups are shown in Fig. 2: the five-year locoregional progression-free survival rates were 82% (95% CI: 62–93) and 83% (95% CI: 65–92), respectively. The difference was not statistically significant (*P* = 0.94).

**Fig. 3.**
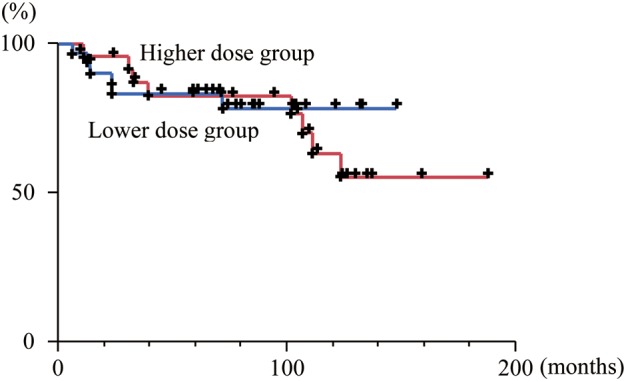
The overall survival curves of the group with higher radiation dose (>30.6 Gy) (red line) and the group with lower radiation dose (≤30.6 Gy) (blue line) among the good responders to chemotherapy.

The overall survival curves for the higher and lower dose groups are shown in Fig. 3: the five-year overall survival rates were 82% (95% CI: 62–93) and 83% (95% CI: 65–92), respectively. The difference was not statistically significant (*P* = 0.73).

## DISCUSSION

A randomized controlled trial conducted by the Southwest Oncology Group (SWOG) showed that three cycles of CHOP followed by IFRT was nearly equivalent to eight cycles of CHOP alone for localized aggressive NHL [1]. In this trial, the radiation dose was not fixed, and ranged from 40–55 Gy, as determined by the individual radiation oncologists. The majority of the patients received a relatively high (>45 Gy) dose. Generally, administration of a higher radiation dose is associated with a higher rate of adverse events. The head and neck area, which is the common origin of aggressive NHL, contains organs that are vulnerable to radiation. Hypothyroidism occurs in 25–50% of patients whose thyroid glands receive irradiation at a dose of 30–40 Gy [3, 17]. A radiation dose of >26 Gy to the salivary gland significantly increases the rate of xerostomia in patients suffering from head and neck malignancies [18]. Moser *et al.* reported that risk of cardiovascular disease was significantly increased in patients receiving a radiation dose >40 Gy as treatment for aggressive NHL [19].

Until recently, the optimal radiation dose for aggressive NHL had not been directly confirmed by a randomized controlled trial. Tsang *et al.* reported that the approaches of the experts in Western countries differed considerably in this regard [20]. A randomized phase III trial was conducted in the UK to verify the effectiveness of reduced dose for local control in NHL [5]. The results of this trial indicated that 30 Gy had the same efficacy as 40–50 Gy in cases of aggressive NHL. These results were convincing due to the large number of patients enrolled in this trial. However, the trial population was relatively heterogeneous. In practice, clinicians are required to make a decision for a patient's specific situation. It is sometimes problematic to adapt the result of a phase III trial, which has some diversity, to such a specific situation [21], and the results of other studies for the specific condition must be taken into consideration. Therefore, we evaluated the validity of a decrease in radiation dose for patients with a specific situation, i.e. localized DLBCL showing a good response to chemotherapy.

There have been several investigations regarding the optimal radiation dose for good responders to chemotherapy. Fuller *et al.* concluded that a dose of ≥40 Gy was needed for these patients to achieve excellent local control [22]. However, other investigators suggested that it was unnecessary to use such a high dose in these cases. Kamath *et al.* reported that 30 Gy was sufficient for good responders to chemotherapy, especially those with non-bulky tumors [7]. Krol *et al.* performed a retrospective study to evaluate the results of combined modality therapy [6]. In their investigation, radiation dose was adopted according to the response to chemotherapy, with good responders to chemotherapy receiving either 26 or 40 Gy; they found no difference in the outcome between patients receiving 26 and those receiving 40 Gy. Similarly, our study did not indicate any benefit of a radiation dose >30 Gy. The results of the present study may yield additional validation for adoption of a decreased radiation dose for good responders to chemotherapy.

The present study had several problems due to its retrospective approach. The lower dose group contained more patients who had undergone R-CHOP than the higher dose group. This may represent a possible bias, but there is no way to validate this. In addition, this study contained other limitations as follows: results from only a single institute, a relatively small sample size, a long study period, and heterogeneity of the treatments.

The efficacy of radiation dose reduction for poor responders to chemotherapy remains a concern. Attending physicians in our facility have consistently administered relatively high doses to patients who have shown a poor response to chemotherapy. Therefore, we were unable to obtain meaningful information about dose reduction for these patients from our database. Further analyses regarding this issue are required.
